# Endotypes of Chronic Rhinosinusitis with Primary and Recurring Nasal Polyps in the Latvian Population

**DOI:** 10.3390/ijms25105159

**Published:** 2024-05-09

**Authors:** Rudolfs Janis Viksne, Gunta Sumeraga, Mara Pilmane

**Affiliations:** 1 Daugavpils Regional Hospital, Vasarnicu Street 20, LV-5417 Daugavpils, Latvia; 2 Department of Doctoral Studies, Riga Stradins University, Dzirciema Street 16, LV-1007 Riga, Latvia; 3 Department of Otorhinolaryngology, Riga Stradins University, Pilsonu Street 13, LV-1002 Riga, Latvia; gunta.sumeraga@rsu.lv; 4 Pauls Stradins Clinical University Hospital, Pilsonu Street 13, LV-1002 Riga, Latvia; 5 Institute of Anatomy and Anthropology, Riga Stradins University, Kronvalda Boulevard 9, LV-1010 Riga, Latvia; mara.pilmane@rsu.lv; 6 Children University Hospital, Vienibas gatve 45, LV-1004 Riga, Latvia

**Keywords:** chronic rhinosinusitis, endotypes, nasal polyps, cytokines, defensins, proliferation marker

## Abstract

Chronic rhinosinusitis (CRS) is a complex syndrome with various inflammatory mechanisms resulting in different patterns of inflammation that correlate with the clinical phenotypes of CRS. Our aim was to use detected IL-1, IL-4, IL-6, IL-7, IL-8, IL-10, IL-12, Ki 67, HBD-2, HBD-3, and LL-37 to classify specific inflammatory endotypes in chronic rhinosinusitis with the tissue of nasal polyps (CRSwNP). Samples from 35 individuals with primary and recurrent CRSwNP were taken during surgery. The tissues were stained for the previously mentioned biomarkers immunohistochemically. A hierarchical cluster analysis was performed. The clinical parameters were compared between clusters. Five clusters had significantly different biomarkers between groups. There were no significant differences in the clinical parameters, except for the Lund–Mackay score, which was significantly higher in cluster 4 compared to that of cluster 1 (*p* = 0.024). Five endotypes of (CRSwNP) are characterized by different combinations of type 1, type 2, and type 3 tissue inflammation patterns. In the Latvian population, endotypes associated with neutrophilic inflammation or a combination of neutrophilic inflammation and type 2 inflammation are predominant. Increased proliferation marker Ki 67 values are not associated with more severe inflammation in the tissue samples of chronic rhinosinusitis with nasal polyps.

## 1. Introduction

Chronic rhinosinusitis (CRS) is a complex syndrome with various inflammatory mechanisms that result in different patterns of inflammation that correlate with the clinical phenotypes of CRS. There are two commonly used phenotypes: chronic rhinosinusitis with nasal polyps (CRSwNP) and chronic rhinosinusitis without nasal polyps (CRSsNP) [1]. The presence of nasal polyps in adult patients is associated with a severe reduction in quality of life, which is usually further compromised by additional comorbidities [2]. Nasal polyps are less common in children, and the corresponding pathogenetic mechanisms differ in the pediatric population. In children, adenoids and genetic factors can lead to the formation of nasal polyps early in life, and the assessment of quality of life in the pediatric population is more challenging [3]. In adults, however, the pathogenesis of CRSwNP is still debated [1].

**Figure 1:**
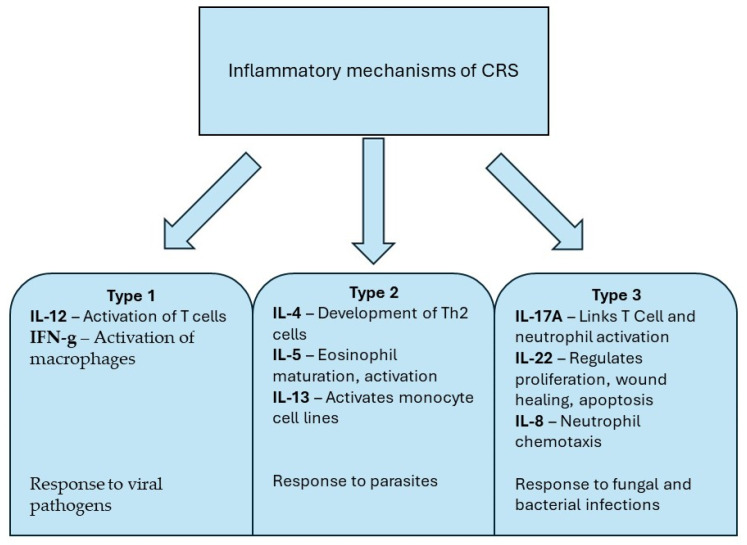
Inflammatory mechanisms of CRS.

A currently researched but yet unproven hypothesis states that different inflammatory mechanisms take place due to dysfunctional interactions at the mucosal level between environmental and host stressors [1]. Sources divide the immunology of inflammation in the airway into Th1 and Th2 subsets. Th1 is primarily related to the viral, fungal, and bacterial defense actions of the host. Th1 and Th17 are dominant, along with type 1 and type 3 innate lymphoid cells. This inflammation pattern is characterized by the generation of antibodies, the activation of macrophages, and the killing of intracellular pathogens. The Th2 inflammation subset, on the other hand, is characterized by Th2 T cell and type 2 innate lymphoid cell activity and increased IgE levels [4]. The European Position Paper on Rhinosinusitis and Nasal Polyps from 2020 describes three general inflammatory mechanisms in CRS. Type 1 inflammation includes factors such as interleukin 12 (IL-12) and interferon-gamma (IFN-g) with responses to viral pathogens. This mechanism involves innate lymphoid cells and dendritic cells. Neutrophil activity and type 1 cytokines may contribute to epithelial barrier damage. The type 2 cytokines are interleukin 4 (IL-4), interleukin 5 (IL-5), and interleukin 13 (IL-13). Interleukin 17A (IL-17A), interleukin 8 (IL-8), and interleukin 22 are type 3 cytokines (Figure 1). Essentially, type 1 reactions fight viruses, type 2 reactions fight parasitic infections, and type 3 reactions fight fungal and bacterial infections [1].

Several studies have further expanded on the idea that there are various inflammatory mechanisms or so-called endotypes by analyzing several biomarkers and using cluster analyses to group several patients with similar biomarker levels in nasal tissue or nasal secretions together in several various endotypes. This statistical approach allows numerous combinations of biomarkers without any perceived outcomes [5].

The cytokines shown in Table 1 are associated with the previously mentioned type 1, type 2, and type 3 mechanisms of inflammation in the nasal tissue in CRS. The proliferation marker Ki 67 is a well-known marker used by histologists to identify cell proliferation [6]. The detection of Ki 67 is important to characterize tissue proliferation and tissue remodulation in each of the inflammatory endotypes. Human beta-defensin-2 (HBD-2), human beta-defensin-3 (HBD-3), and cathelicidin LL 37 (LL-37) are small peptides with potent antimicrobial properties. HBD-2 is a potent antimicrobial peptide that kills Gram-negative bacteria. LL 37 and HBD-3 actively kill Gram-negative and Gram-positive bacteria [7]. These antimicrobial peptides are important factors that could help in associating specific endotypes with possible pathogeneses linked to bacterial agents. Currently, the CRS pathogenesis hypothesis states that an impaired epithelial barrier function can cause chronic inflammation wherein type 1, type 2, and type 3 pathways could be active in combinations or alone [1]; however, further research is necessary.

Therefore, our aim was to use detected IL-1, IL-4, IL-6, IL-7, IL-8, IL-10, IL-12, Ki 67, HBD-2, HBD-3, and LL-37 to classify specific inflammatory endotypes in chronic rhinosinusitis with the tissue of nasal polyps (CRSwNP).

## 2. Results

**Figure 2:**
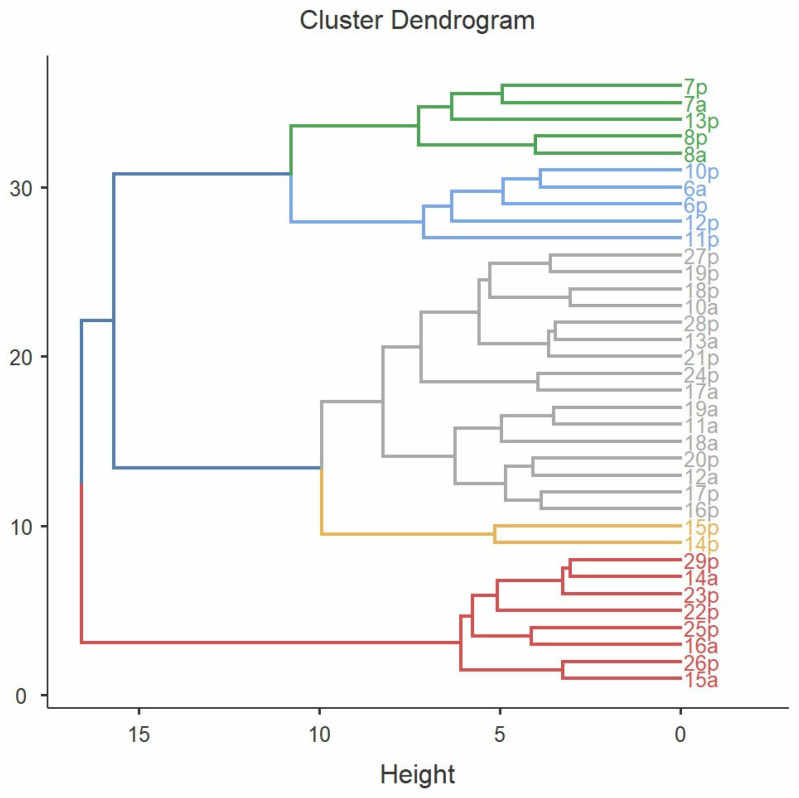
The division of patients into separate clusters.

Hierarchical cluster analysis revealed five distinctive clusters or inflammatory endotypes based on the analyzed biomarkers (Table 2). The dendrogram below depicts individual patients and their grouping into similar clusters (Figure 2).

**Figure 3:**
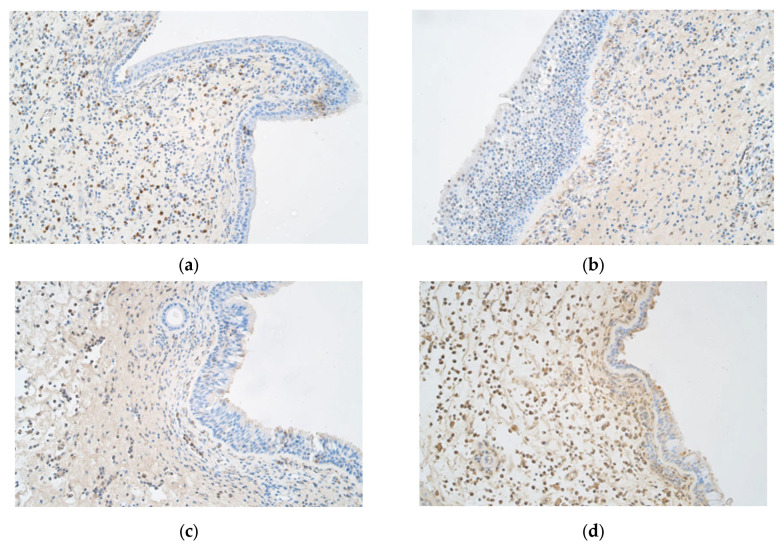
(**a**–**d**) Immunohistochemical micrographs of patients with nasal polyps. (**a**) Polyp sample with occasional to few IL-1α-positive structures in the epithelium and numerous positive structures in the connective tissue—IL-1α IMH, ×200. (**b**) Note the occasional IL-4-positive structures in the epithelium as well as the moderate number of positive structures in the connective tissue of nasal polyp mucosa—IL-4 IMH, ×200. (**c**) The nasal polyp sample has few IL-6-positive structures in the epithelium but a moderate number of positive structures in the connective tissue—IL-6 IMH, ×200. (**d**) Note the few to moderate number of IL-7-positive structures in the epithelium and the numerous positive structures in the connective tissue—IL-7 IMH, ×200.

**Figure 4:**
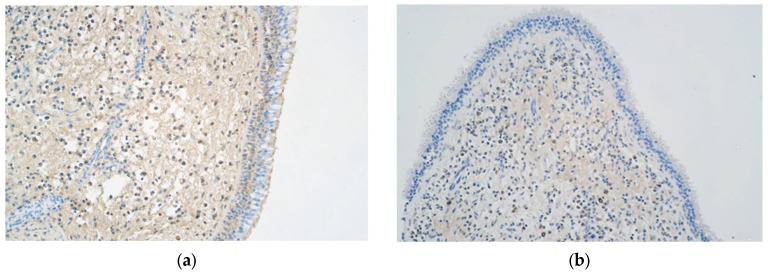

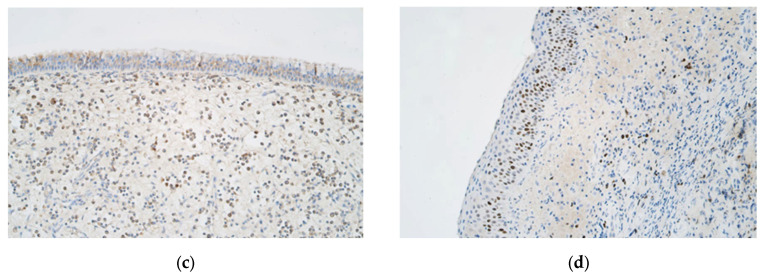
(**a**–**d**) Immunohistochemical micrographs of patients with nasal polyps. (**a**) Polyp sample with moderate IL-8-positive structures in the epithelium and numerous positive structures in the connective tissue (IL-8 IMH, ×200). (**b**) Note the occasional IL-10-positive structures in the epithelium as well as the moderate number of positive structures in the connective tissue of this nasal polyp sample (IL-10 IMH, ×200). (**c**) This nasal polyp sample has a moderate number of IL-12-positive structures in the epithelium but numerous positive structures in the connective tissue (IL-12 IMH, ×200). (**d**) Note the moderate number of Ki 67-positive structures in the epithelium of this polyp sample and the few to moderate number of positive structures in the connective tissue (Ki 67 IMH, ×200).

When examining the immunohistochemical graphs of the nasal polyp samples, certain characteristics were observed. First, the polyp samples showed vast subepithelial connective tissue with little to no submucosal glandular tissue. Subepithelial connective tissue often showed a much larger number of positive structures than the epithelial layer (Figure 3 and Figure 4).

The heat map depicted in Table 2 shows differences between the average number of positive structures in each cluster, where dark green represents the highest values, light green indicates the intermediate values, and white cells show the lowest values in each row.

### 2.1. Proliferation Marker Ki 67

This marker (shown in Figure 4d) was found in significantly greater proportions in the epithelial cells of cluster 1 patients than in any of the other clusters (*p* = 0.030). It showed intermediate values in clusters 2 and 5, but clusters 3 and 4 had the lowest number of positive structures in the epithelium. In the connective tissue, there were no significant differences between all the clusters regarding Ki 67; yet, when compared individually, there were significantly more Ki 67-positive structures in the connective tissue in cluster 1 than in clusters 3 (*p* = 0.005), 4 (*p* = 0.27), and 5 (*p* = 0.34).

### 2.2. Interleukin 1α

The levels of this cytokine (Figure 3a) were found to be significantly higher in the epithelium (*p* < 0.001) and subepithelial connective tissue (*p* = 0.007) in cluster 2 compared to those in the other clusters (Table 2).

### 2.3. Interleukin 4

The levels of this cytokine (Figure 3b) were found to be significantly higher in the subepithelial connective tissue in the cluster 5 samples in comparison to those in the other clusters (*p* = 0.001). In the epithelium, there were no major differences between clusters; yet, individually, there were significant differences between clusters 5 and 3 (*p* = 0.043) and between clusters 5 and 4 (*p* = 0.029) (Table 2).

### 2.4. Interleukin 6

The levels of interleukin 6 (Figure 3c) were significantly higher in the epithelium in the cluster 2 samples (*p* = 0.018), but there were no significant differences between clusters in the connective tissue of the samples (*p* = 0.098); however, individually, there was a significant difference between clusters 2 and 5 (*p* = 0.019) (Table 2).

### 2.5. Interleukin 7

There were no significant differences in epithelial cells (*p* = 0.058) when evaluating interleukin 7 (Figure 3d), but in the case of the connective tissue, cluster 4 showed significantly fewer positive structures than the other clusters (*p* = 0.006) (Table 2).

### 2.6. Interleukin 8

For this cytokine (Figure 4a), a significant increase was observed between cluster 3 and the other clusters (*p* = 0.019), except cluster 5. In the connective tissue, IL-8 levels showed a significant increase in cluster 3 and cluster 5 compared to those in the other clusters (*p* < 0.001) (Table 2).

### 2.7. Interleukin 10

This interleukin (Figure 4b) demonstrated a significant increase in the number of positive epithelial structures in cluster 1 (*p* = 0.004); conversely, in the connective tissue, cluster 3 exhibited the highest increase compared to the other clusters (*p* < 0.001) (Table 2).

### 2.8. Interleukin 12

IL-12 was higher in all the connective and epithelial tissue samples, except those in cluster 4, where both the epithelial (*p* < 0.001) and connective tissues (*p* = 0.005) showed a significant decrease (Table 2).

### 2.9. Human β-Defensin-2

**Figure 5:**
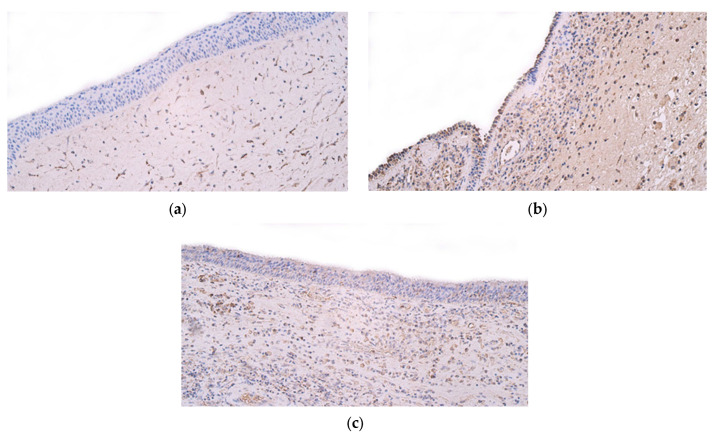
(**a**–**c**) Immunohistochemical micrographs of patients with nasal polyps. (**a**) Polyp sample with no β-defensin-2-positive structures in the epithelium and few positive structures in the connective tissue (β-defensin-2 IMH, ×200). (**b**) Note the numerous β-defensin-3-positive structures in the epithelium as well as the moderate to large number of positive structures in the connective tissue in this nasal polyp sample (β-defensin-3 IMH, ×200). (**c**) This nasal polyp sample has a small to moderate number of LL-37-positive structures in the epithelium but numerous positive structures in the connective tissue (β-defensin-3 IMH, ×200).

When we evaluated this antimicrobial protein in our samples (Figure 5a), in the epithelium, its levels were higher in cluster 1 compared to those in clusters 2, 3, and 4 (*p* = 0.010). There were no significant differences between cluster 5 and the other clusters. In the connective tissue, the number of structures was equally divided among the clusters, except cluster 4, which showed a significant decrease (*p* = 0.045) (Table 2).

### 2.10. Human β-Defensin-3

β-defensin (Figure 5b) was found in significantly greater numbers in epithelial cells in cluster 5 than in any other cluster (*p* < 0.001), but in the connective tissue, these values were equal, with the exception of cluster 3, which contained samples with significantly more positive structures than cluster 4 (*p* = 0.016), and cluster 2, which exhibited a significant decrease in connective tissue-positive structures compared to the other clusters (*p* = 0.004) (Table 2).

### 2.11. Cathelicidin LL 37

This antimicrobial peptide (Figure 5c) was found in significantly greater numbers in the connective tissue of the cluster 3 and cluster 5 samples (*p* < 0.001) compared to those of the other clusters. There were no significant differences between clusters in terms of the epithelial samples, but cluster 5 had significantly more positive epithelial structures than cluster 1 (*p* = 0.038) and cluster 4 (*p* = 0.021) when compared individually with one another (Table 2).

Several clinical parameters, such as the presence of bronchial asthma, allergies, active smoking, and history of long-term smoking, were evaluated for each cluster of patients. There were no significant differences observed between groups (Table 3). Yet, there were individual differences. The Lund–Mackay score was significantly increased in cluster 4 compared to that of cluster 1 (*p* = 0.024).

Previous conservative treatments received prior to surgery were documented. The use of intranasal corticosteroids, courses of peroral corticosteroids, and long courses of macrolide antibiotic use in the year leading up to the surgery were documented. There were no significant differences between conservative treatments received before surgery (Table 4).

### 2.12. Endotype Differences

The most common endotype is the third one, characterized by moderate IL-4 levels, increased IL-8 levels, and connective tissue antimicrobial proteins characterized by a combination of type 2 and type 3 inflammation patterns.

The second most common is the fourth endotype, which is characterized by a decrease in all markers except IL-8, HBD-3, and LL 37 and shows an isolated type 3 inflammation pattern.

The third most common endotype is the fifth endotype, which consists of increased type 2 and type 3 inflammatory patterns and a strong presence of antimicrobial proteins with a clinical association with long-term smoking.

The second least common endotype is a combination of type 1 and type 2 inflammation patterns, which demonstrates increased IL-1α, IL-6, and IL-12 levels with a moderate increase in IL-4 levels and more aggressive inflammation.

The first endotype is also less common and is characterized by increased Ki 67, IL-12, and HBD-2 presence, a slow clinical progression, and type 1 and type 2 inflammation patterns.

## 3. Discussion

In our samples of primary and recurrent nasal polyps, we observed large amounts of subepithelial connective tissue and edema. The subepithelial glandular tissue was mostly absent or underdeveloped. Large quantities of positive structures of our analyzed biomarkers were found in the subepithelial connective tissue as opposed to the epithelial tissue, where fewer positive structures were found. We discussed the distribution of these very biomarkers in our previous publications, where we analyzed the distributions of cytokines, proliferation marker Ki 67, and antimicrobial and defense proteins in the tissues of recurrent and primary nasal polyps and compared them to the control samples of healthy nasal mucosa. Significant differences were found, showing decreased biomarker presence in epithelial cells and increased presence in the subepithelial connective tissue of nasal polyps compared to the control samples [14,15,16]. With the addition of clinical parameters, we were able to characterize five different clusters of patients with similar individual distributions of biomarkers.

### 3.1. The First Cluster

Cluster 1 demonstrated increased Ki 67 concentrations in epithelial and connective tissues; these levels were much higher than those in the other clusters. Ki 67 is a marker used to treat various cancer types to estimate prognosis and influence further treatment options [17]. In addition to malignant processes, Ki 67 is also present in tissues with severe inflammation [18]. Ki 67 is useful for detecting cell proliferation [6]. Its presence could be an indicator that this specific cluster is characterized by more proliferative changes and tissue remodulation. In addition, intermediate levels of IL-6 could be associated with cell proliferation due to increased Ki 67 levels, as hypothesized in a study by Bequignon et al., where IL-6 was considered to be a possible inducer of cell proliferation in nasal polyps [10]. Cluster 2 showed markedly increased IL-6 levels and relatively decreased Ki 67 values, meaning that although IL-6 might be linked to cell proliferation in one endotype, it might not be as important in all inflammatory endotypes, as we thought in our previous research [16]. Interestingly, IL-12 displayed a significant increase in this cluster. IL-12 helps T cells to differentiate into Th1 cells. It is associated with the activity of T cells as well as NK cells [19]. IL-12 is associated with type 1 inflammation in chronic rhinosinusitis [1]. It is hypothesized that IL-12 and IL-4 balance the activity of type 1 and type 2 lymphocytes in CRSwNP [20]. An increase in epithelial IL-4 levels along with an intermediate increase in connective tissue IL-4 levels could be associated with a balance between type 1 and type 2 inflammation patterns in this specific endotype. This cluster also showed an increase in human β-defensin-2 levels both in epithelium and connective tissues. HBD-2 is normally found in the epithelial cells of nasal mucosa. In cases of chronic inflammation, it can be upregulated [21]. An increase in the levels of this peptide in the subepithelial connective tissue might suggest a dysfunction of the epithelial barrier and the presence of bacterial pathogens in the subepithelial connective tissue. HBD-3 levels were also found to be higher in the subepithelial connective tissue. HBD-3 and HBD-2 both have potent antimicrobial activity against Gram-negative bacteria such as Pseudomonas aeruginosa and Escherichia coli. Additionally, HBD-3 actively kills Gram-positive bacteria like Staphylococcus aureus [22]. In regard to our findings, this could mean that S. Aureus might be involved in the inflammation processes in this cluster. Overall, this cluster demonstrates type 1 and type 2 inflammation patterns; yet, the decreased IL-8 values observed demonstrate that active neutrophil recruitment does not take place as actively as it does in the other clusters. IL-10 is a regulatory cytokine that possesses the ability to downregulate inflammation [23]. In this cluster, it was expressed more in the epithelial tissue in comparison to the other clusters, meaning that the immune system actively downregulated inflammatory effects at the epithelial level. Only one of the patients from this cluster was experiencing a recurrence of nasal polyps after a previous surgery that took place 23 years prior. In addition, cluster 1 demonstrated a significantly lower Lund–Mackay score than cluster 4 and lower, although nonsignificant, SNOT 22 scores than the other clusters, categorizing this endotype as less aggressive than the others.

### 3.2. The Second Cluster

Cluster 2 had moderate levels of Ki 67-positive structures compared to the other clusters, meaning proliferation might not have been very significant but was still present. All the patients in this cluster had a diagnosis of bronchial asthma. In this cluster, we observed significantly increased levels of IL-1α in the epithelium as well as the connective tissue. IL-1α is a proinflammatory cytokine that is secreted from cells early and starts initial localized inflammatory reactions. IL-1α is stored in cells in an active state and, if released when a cell dies, functions as an alarmin [24]. Given the increased levels of this cytokine, we can assume that this cluster has more active inflammation as well as greater potential cell destruction both in epithelium and connective tissues than the other clusters. This can also be supported by the rather increased levels of IL-6 that, if Bequignon et al.’s previously mentioned hypothesis of IL-6-mediated epithelial barrier damage is correct, could explain increases in IL-1α. Surprisingly, antimicrobial protein values were not significantly higher than those of other clusters; in fact, there were no positive HBD-3 structures found in any of the subject samples. Only intermediate values of connective HBD-2 and epithelial LL 37 were observed, raising the question of whether the lack of antimicrobial peptides might be the cause of tissue cell damage and active inflammation. Of course, we need to consider that smoking can reduce HBD-2 as well as IL-8 levels in sinonasal tissue and that three out of four patients in this cluster had a history of long-term smoking [25]. On the other hand, a different study suggested that cigarette smoke can reduce HBD-2 production but enhance IL-8 production [26]. A complete lack of HBD-3-positive structures could either mean that there is no direct relation to Gram-positive bacteria like S. Aureus in this cluster or a compromised antimicrobial response to these kinds of bacteria. Increased IL-1α levels along with increased IL-6 levels might characterize this cluster as having more active inflammation than others.

### 3.3. The Third Cluster

Cluster 3 is where most of our subject samples fell into, meaning that this might be the most common inflammatory endotype in the Latvian population. More than half of the cluster patients were experiencing a recurrence of nasal polyps after previous surgery. This cluster had the largest number of IL-8 epithelial and connective tissue-positive structures. IL-8 is a very important chemotactic factor that induces chemotaxis in neutrophils and other granulocytes towards an infection site [27]. It can be associated with a type 3 inflammation pattern as it is an important factor for neutrophil recruitment [1]. Overall, cluster 5 and cluster 3 displayed similar biomarker patterns that are characteristic of a type 3 reaction pattern; yet, cluster 5 also exhibited an additional type 2 pattern. Cluster 3 also demonstrated a significantly greater number of expressed IL-10 in the subepithelial connective tissue, indicating active attempts of the immune system to downregulate inflammation, which contrasts with the case for cluster 1, where IL-10 activity was more expressed in the epithelium.

### 3.4. The Fourth Cluster

Cluster 4 demonstrated the lowest levels of every biomarker compared to the other clusters. It only showed intermediate levels of connective tissues IL-8, LL 37, and HBD-3, meaning that these cases could be exclusively associated with a type 3 inflammation pattern and possible subepithelial tissue infection with S. Aureus. This can be further supported by the fact that LL 37 is present in intermediate levels in the subepithelial connective tissue and possesses activity against S. Aureus [28]. The presence of LL 37 can also be associated with the presence of neutrophil extracellular traps whose increased presence in the tissue of nasal polyps is a recent novel finding [29].

### 3.5. The Fifth Cluster

Cluster 5 demonstrated high levels of type 2 inflammatory cytokine IL-4 as well as neutrophil chemotaxis factor IL-8 associated with the type 3 pattern. Type 3 and type 2 inflammatory pathways can often co-exist, which implies that there could be a superimposed reaction to microbiota in patients with chronic rhinosinusitis [1]. In this case, all the patients were long-time smokers, but HBD-2 and IL-8 levels were not significantly lower, as previously discussed. The patients also showed a significantly higher increase in HBD-3 and LL 37 levels, leading us to believe that along with moderate values in HBD-2 positive structures, we can see a combined type 2 and type 3 reaction pattern in response to the possible disruption of the epithelial barrier and polymicrobial presence in the tissue of nasal polyps. Also, as mentioned before, an increase in LL 37 and IL-8 could potentially indicate the formation of neutrophil extracellular traps.

### 3.6. Other Studies on CRS Endotypes

A recent study by Wang et al. evaluated forty-eight inflammatory and remodeling factors in the tissue samples of patients with CRSwNP and CRSsNP by using cluster analysis. The results showed that there were five different clusters with various degrees of the presence of bronchial asthma, allergies, and recurrences. The results also revealed that cluster 1 and cluster 2 had low type 2 reactions, cluster 3 had a low type 2 pattern and high expression of neutrophil factors such as IL-8, cluster 4 had a high type 2 pattern, and cluster 5 had high type 2 inflammation and neutrophil and remodeling factors [30]. Our findings, although only associated with CRSwNP, have some similar patterns. For example, our cluster 3 demonstrated high concentrations of IL-8, which is associated with neutrophilic inflammation; similarly, cluster 5 demonstrated high levels of type 2 pattern and neutrophilic inflammation, and clusters 2 and 1 similarly showed moderate type 2 inflammation. In 2018, Turner et al. performed cluster analysis using 18 biological variables [31]. They found six clusters with variable clinical presentation of asthma and nasal polyps. Their findings for cluster 6 were similar to our findings for cluster 1: their cluster also had increased levels of IL-4, IL-6, IL-7, and IL-12; however, it was considered a high-inflammation cluster, while ours was not. Other clusters also presented some similarities. Liao et al. evaluated tissue samples and used 28 clinical and 39 mucosal variables for principal component analysis and performed cluster analysis [32]. They found seven clusters with different biological marker characteristics. As in our findings, they showed a cluster with nasal polyps and relatively low concentrations of biological markers, like our cluster 4. They also presented a cluster with high type 2 inflammation, a cluster with high IL-12 values, and another cluster with increased IL-8 and neutrophil quantities. Tomassen et al. selected 14 biological markers for tissue analysis, used partition-based clustering, and produced 10 different clusters [33]. Although the cluster counts differ significantly, there are similar patterns to those in our work. The last 10 of the clusters demonstrate type 2 and type 3 patterns like our cluster 5; there are intermediate clusters with increased type 2, type 3, and type 1 characteristic biomarkers, and there are more clinically less aggressive clusters with type 1 factors such as type 1 marker INF-γ and increased IL-6, like our cluster 1 and cluster 2. Interestingly, in a recent study in Korea, five clusters of patients with chronic rhinosinusitis were found, and the most common finding was a type 3 endotype with a high presence of nasal polyps, severe disease extent, and type 2 CRS [34].

### 3.7. Treatment Received

When we evaluated the treatment methods received prior to surgery, there were no significant differences between groups, although more patients received courses of peroral corticosteroids and antibiotics in cluster 3. This finding might be associated with a more aggressive presentation of clinical symptoms, thus requiring clinicians to try more conservative treatment options as it was seen that more than half of the patients in cluster 3 experienced a recurrence of symptoms. The use of intranasal corticosteroids remained the most common treatment option among all clusters. We found no significant associations between the conservative treatment and tissue factor changes. In the future, understanding the inflammatory patterns and endotypes of patients could help in tailoring individual endotype-based treatments using biological compounds. Biological medication is a promising addition that could help reduce the need for surgery [35]. Our study gives insights into the specific endotypes of the population of Latvia and, in the future, could be used to individualize treatments.

### 3.8. Limitations

Our study has some limitations. If possible, we would have used a larger cohort for this study. Although the analysis of various immunohistochemical markers among a smaller amount of tissue samples is widely accepted in morphological studies, we would be able to present more precise clinical results with a much larger sample group. Therefore, future studies with a larger sample size would be beneficial, although, in our opinion, they would not change the basic characteristics of each cluster. Various types of methods for assessing the presence of biomarkers, such as ELISA, in situ hybridization, and gene detection, would be beneficial in our future research.

Also, the consideration of other factors, such as CCL3 or macrophage inflammatory protein 1α, could be beneficial to account for geographical population variability in the case of CRS endotypes [36].

## 4. Materials and Methods

The study group consisted of 35 (26 male and 9 female) patients. The inclusion criteria for the study group were a diagnosis of CRSwNP made by a certified otolaryngologist and having been scheduled for an endoscopic sinus surgery. The exclusion criteria consisted of coagulopathies, immunodeficiencies, or an exacerbation of CRS symptoms 2 weeks prior to surgery. The mean age of the patients was 48.3 (±14.2) years. The study group was further divided into 22 patients undergoing their first sinus surgery and 13 patients undergoing repeated surgery after a recurrence of nasal polyps. Samples of nasal polyps were taken during planned functional endoscopic sinus surgery (FESS). As for the control samples, we previously published and characterized control group samples taken from the healthy nasal mucosa of patients undergoing septoplasty in our previous studies [14,15,16]. The research proposal was reviewed and approved by the ethics committee of Riga Stradins University (6-1/10/59. 26 October 2020). The nature of this study was explained, and the patients gave informed consent. The samples were put in a mixture of 2% formaldehyde and 0.2% picric acid in a 0.1 M phosphate buffer (pH 7.2) for up to 72 h, rinsed in Tyrode buffer (content: NaCl, KCl, CaCl_2_·2H_2_O, MgCl2·6H_2_O, NaHCO_3_, NaH_2_PO_4_·H_2_O, and glucose) containing 10% saccharose for 12 h, and then embedded into paraffin. Biotin-streptavidin was used as an immunohistochemical method for the detection of IL-1α (orb308737, 1:100, Biorbyt, Cambridge, UK); Ki 67 (1325506A, 1:100, Cell Marque, Rocklin, CA, USA); IL-4 (orb10908, 1:100, Biorbyt, Cambridge, UK); IL-7 (orb13506, 1:100, Biorbyt, Cambridge, UK); IL-6 (sc-130326, 1:100, Santa Cruz Biotechnology Inc., Dallas, TX, USA); IL-8 (orb39299, 1:100, Biorbyt); IL-10 (250713, 1:100, BioSite, Täby, Sweden); IL-12 (orb10894, 1:100, Biorbyt); HBD-2 (sc-20798, working dilution—1:100, Santa Cruz Biotechnology, Inc., Dallas, TX, USA); HBD-3 (rb183268, working dilution—1:100, Biorbyt Limited, Cambridge, UK); and LL 37 (orb88370, working dilution—1:100, Biorbyt Limited, Cambridge, UK). The slides were evaluated using light microscopy in conjunction with the semi-quantitative counting method to detect positively stained cells in the visual field. The number of positive structures was assessed using a semi-quantitative method [37]. The values obtained using the semi-quantitative method as well as the numbers into which they were transformed are depicted in Table 5.

It is a widely accepted practice to represent the number of positive structures as symbolic values such as ‘’+’’ and to transform them into numbers for statistical purposes in morphological studies [38]. Statistical analysis was performed using IBM SPSS software version 26 and Jamovi software version 2.4.11. Principal component factor analysis was performed using Varimax rotation with Kaiser normalization, and only variables with loadings greater than 0.4 were included. The number of clusters was determined by assessing the number of components with an eigenvalue greater than 1 and cumulative factor variance that explained at least 70% of the variance. The adequacy of the samples was measured by excluding variables with a correlation quotient with a value less than 0.5. Also, Bartlett’s test of specificity was used. In addition, the bend on a scree plot was used to determine the optimal number of components. A hierarchical cluster analysis was performed using the Ward method with squared Euclidian distances with the standardization of values using z scores. In addition, the Mann–Whitney U and Kruskal–Wallis H tests were used to assess significant differences between two factors and between variables in a group. Also, a chi-squared test and a Fisher’s exact test were used.

## 5. Conclusions

Five endotypes of chronic rhinosinusitis with nasal polyps are characterized by different combinations of type 1, type 2, and type 3 tissue inflammation patterns. In the Latvian population, endotypes associated with neutrophilic inflammation or a combination of neutrophilic inflammation and type 2 inflammation are predominant. Increased proliferation marker Ki 67 values are not associated with more severe inflammation in tissue samples of chronic rhinosinusitis with nasal polyps.

## Figures and Tables

**Table 1:** Descriptions of cytokines.

Interleukin	Description
Interleukin 1 alpha (IL-1α)	It is a proinflammatory cytokine. IL-1 derives from the epithelium and can directly and indirectly control type 2 cytokine production by epithelial cells [8].
Interleukin 4 (IL-4)	It is also a proinflammatory cytokine. IL-4 is one of the key cytokines in type 2 inflammation. IL-4 stimulates CD4+ cell polarization to Th2 cells, and it is also essential in the functioning of normal healthy mucosa [4].
Interleukin 6 (IL-6)	This cytokine plays an important role in chronic inflammation and autoimmunity [9]. IL-6 has also been associated with epithelial barrier dysfunction and the pathogenesis of CRS [10].
Interleukin 7 (IL-7)	IL-7 is an important factor in the survival of naïve T cells, T memory cells, pro B cells, and innate lymphocytes [11].
Interleukin 8 (IL-8)	IL-8 is an important factor for neutrophil recruitment and chemotaxis and is therefore associated with type 3 inflammation in CRS [12].
Interleukin 10 (IL-10)	It is a cytokine with an anti-inflammatory function. Upon encountering allergens, epithelial cells release IL-10 to downregulate type 2 inflammatory effects as well as other inflammatory mechanisms [13].
Interleukin 12 (IL-12)	It is a proinflammatory cytokine associated with type 1 inflammation in CRS [1].

**Table 2:** A heat map showing the average number of positive structures in each cluster.

	Cluster 1	Cluster 2	Cluster 3	Cluster 4	Cluster 5	*p* Value
Ki 67 E	1.375	0.750	0.167	0.056	0.583	0.030
Ki 67 CT	1.125	0.500	0.208	0.389	0.250	0.054
IL-1 E	0.875	1.875	0.417	0.167	0.750	<0.001
IL-1 CT	1.500	2.500	1.667	1.167	1.917	0.007
IL-4 E	1.500	1.250	1.042	0.889	1.583	0.070
IL-4 CT	2.125	2.125	1.833	1.389	3.250	0.001
IL-6 E	1.250	1.500	0.917	0.722	1.000	0.018
IL-6 CT	2.250	2.625	2.417	1.833	1.917	0.098
IL-7 E	1.125	1.750	1.125	0.667	1.333	0.058
IL-7 CT	2.500	2.500	2.583	1.500	2.333	0.006
IL-8 E	0.500	0.625	1.042	0.444	1.167	0.019
IL-8 CT	1.125	1.000	2.667	1.556	2.083	<0.001
IL-10 E	1.250	0.500	0.458	0.056	0.333	0.004
IL-10 CT	1.500	1.125	2.708	1.611	1.667	<0.001
IL-12 E	2.375	2.500	1.250	0.778	1.583	<0.001
IL-12 CT	3.250	2.875	2.792	1.667	2.750	0.005
Bdef2 E	0.625	0.000	0.083	0.000	0.250	0.010
Bdef2 CT	1.125	1.125	1.375	0.278	1.250	0.045
Bdef3 E	0.250	0.000	0.500	0.056	1.167	<0.001
Bdef3 CT	1.750	0.000	2.208	1.167	2.000	0.004
LL 37 E	0.250	0.875	0.708	0.389	1.250	0.058
LL 37 CT	0.625	0.750	2.417	1.278	2.583	<0.001

Abbreviations: Ki 67—proliferation marker; IL-1—interleukin 1; IL-4—interleukin 4; IL-6—interleukin 6; IL-7—interleukin 7; IL-8—interleukin 8; IL-10—interleukin 10; IL-12—interleukin 12; Bdef2—Human beta-defensin-2; Bdef3—Human beta-defensin-3; LL 37—cathelicidin LL 37; E—epithelial tissue; and CT—subepithelial connective tissue. Dark green—highest values in the row; Light green—average values in the row; White—lowest values in the row.

**Table 3:** Clinical parameters of each cluster.

	Cluster 1	Cluster 2	Cluster 3	Cluster 4	Cluster 5
Number of individuals	4	4	12	9	6
Average age	52.5 (±8.26)	41.5 (±9.95)	49 (±18.64)	49.3 (±12.85)	47.2 (±13.89)
Lund–Mackay score	13.25 (±0.96)	18.25 (±5.06)	16.00 (±6.52)	19.44 (±3.88)	16.67 (±4.08)
Snot 22 score	28.25 (±12.99)	46.50 (±19.34)	46.08 (±24.62)	37.44 (±18.17)	47.67 (±32.32)
History of allergies	1/4	2/4	6/12	2/9	1/6
Bronchial asthma	1/4	4/4	6/12	4/9	1/6
Active smoker	1/4	1/4	1/12	2/9	3/6
History of long-term smoking	2/4	3/4	7/12	6/9	6/6
Previous surgery	1/4	2/4	7/12	3/9	0/6
Average number of years passed since last surgery	23 (±0)	7.5 (±9.19)	5.7 (±6.21)	4 (±2.65)	

**Table 4:** Conservative treatments prior to surgery.

	Cluster 1	Cluster 2	Cluster 3	Cluster 4	Cluster 5
Intranasal corticosteroids	2/4	4/4	7/12	7/9	6/6
Oral corticosteroids	2/4	1/4	5/12	2/9	0/6
Long course of antibiotics	2/4	1/4	4/12	0/9	1/6

**Table 5:** Semi-quantitative counting method.

Positive Structures in the Visual Field	Expressed as Symbols	Expressed as Numbers
No structures	0	0
Occasional	0/+	0.5
Few	+	1
Moderate	++	2
Numerous	+++	3
Abundant	++++	4

## Data Availability

The data presented in this study are available upon request from the corresponding author.

## Abbreviations

CRS	Chronic rhinosinusitis
CRSsNP	Chronic rhinosinusitis without nasal polyps
CRSwNP	Chronic rhinosinusitis with nasal polyps
HBD-2	Human β-defensin-2
HBD-3	Human β-defensin-3
IL-1α	Interleukin 1α
IL-10	Interleukin 10
IL-12	Interleukin 12
IL-13	Interleukin 13
IL-17A	Interleukin 17A
IL-4	Interleukin 4
IL-6	Interleukin 6
IL-7	Interleukin 7
IL-8	Interleukin 8
Ki 67	Proliferation marker Ki 67
LL-37	Cathelicidin LL 37
